# Evaluation of Optimized Tube-Gel Methods of Sample Preparation for Large-Scale Plant Proteomics

**DOI:** 10.3390/proteomes6010006

**Published:** 2018-01-30

**Authors:** Thierry Balliau, Mélisande Blein-Nicolas, Michel Zivy

**Affiliations:** PAPPSO, GQE-Le Moulon, INRA, Univ. Paris-Sud, CNRS, AgroParisTech, Paris-Saclay University, 91190 Gif-sur-Yvette, France; thierry.balliau@inra.fr (T.B.); melisande.blein-nicolas@inra.fr (M.B.-N.)

**Keywords:** plant proteomics, protein extraction, protein digestion, tube-gel, FASP, shotgun proteomics

## Abstract

The so-called tube-gel method is a sample preparation protocol allowing for management of SDS for protein solubilization through in-gel protein trapping. Because of its simplicity, we assumed that once miniaturized, this method could become a standard for large scale experiments. We evaluated the performances of two variants of the miniaturized version of the tube-gel method based on different solubilization buffers (Tris-SDS or urea-SDS). To this end, we compared them to two other digestion methods: (i) liquid digestion after protein solubilization in the absence of SDS (liquid method) and (ii) filter-aided sample preparation (FASP). As large-scale experiments may require long term gel storage, we also examined to which extent gel aging affected the results of the proteomics analysis. We showed that both tube-gel and FASP methods extracted membrane proteins better than the liquid method, while the latter allowed the identification and quantification of a greater number of proteins. All methods were equivalent regarding quantitative stability. However, important differences were observed regarding post-translational modifications. In particular, methionine oxidation was higher with the tube-gel method than with the other methods. Based on these results, and considering time, simplicity, and cost aspects, we conclude that the miniaturized tube-gel method is suitable for sample preparation in the context of large-scale experiments.

## 1. Introduction

Sample preparation for proteomics analyses is crucial, since it determines the quality of the final results. The first step is protein extraction. It determines which population of proteins will be analyzed, and whether the integrity of these proteins, including their post-translational modifications (PTMs), will be conserved. In bottom-up shotgun experiments, protein extraction is directly followed by protein digestion, the efficiency of which depends on the sample composition (solvents, detergents, chaotrops). The last step is mass spectrometry (MS) analysis, which is incompatible with the presence of sodium dodecyl sulfate (SDS) in the final peptide mixture. Yet, SDS is of one of the most powerful detergents, useful for the extraction of proteins in general, and membrane proteins in particular.

Several methods of sample preparation have been developed to remove SDS before MS analysis. The simplest one consists in not using SDS during protein extraction, and to carry out in-solution digestion. However, in this case, protein extraction is not performed in optimal conditions. Alternative methods exist based either on the exchange of SDS by urea after concentration of the proteins on a filter (filter-aided sample preparation, FASP [1]), or on in-gel digestion of proteins. In the latter case, samples are electrophorized (SDS-PAGE), and proteins are precipitated within the gel. After a succession of rinsing baths to eliminate SDS and other unwanted compounds, proteins are digested directly in-gel. In 2005, Lu et al. [2] simplified this gel-based method by removing the electrophoresis and protein precipitation steps. Their method, called tube-gel, indeed consists in mixing proteins with an acrylamide solution so that they are subsequently trapped in the gel matrix during the polymerization process. SDS is then removed by extensive gel cleaning. Several authors used the tube-gel method for their analyses (e.g., [3,4]). Some also showed that it can be used in shotgun experiments, including for quantitative comparisons [5], and others developed variants (e.g., gel-aided sample preparation [6]).

Although it has been developed specifically for membrane proteins, we assumed that the tube-gel method could become a standard to analyze total proteins in the framework of large-scale experiments. Indeed, an increasing number of omics studies require the analysis of a large number of samples, especially in quantitative genetics and in systems biology. Several proteomics articles have already been published, that report the analysis of more than a hundred of samples to study the natural genetic variability [7,8], to search for candidate proteins for complex traits [9], to analyze the determinism of protein heredity [10], or to construct regulatory networks in systems biology experiments [11]. Preparing a high number of samples for MS analysis is tedious and costly. Quick and cheap protocols can be used, but at the expense of data quality. Because of its simplicity, its ability to manage SDS, and its low cost in terms of consumables, the tube-gel method could represent a good compromise between the cost and time of sample handling on one side, and data quality on the other side. 

To evaluate this possibility, we developed a miniaturized version of the tube-gel method to make it as convenient as possible for large-scale use. We tested two variants of this method based on different SDS-containing buffers for protein solubilization: one contained Tris (tube-gel-T method) and the other contained urea (tube-gel-U method). As the preparation of a large number of samples is time-consuming, we also tested whether long-term storage of in-gel proteins affected the results of the MS analysis. To evaluate whether the quality of the data obtained after method optimization was acceptable, we compared the performances of the tube-gel variants to those of two commonly used methods less adaptable to high throughput: an in-solution digestion method (named liquid method in the following), which is our present standard method, and the FASP method, considered here as a standard for the analysis of membrane proteins. For proper comparisons, we used the same starting protein extract obtained from maize leaves, and the same amounts of protein and trypsin for digestion. Methods were evaluated on the criteria of extraction yield, protein identification, PTMs, identification of membrane proteins, and quantitative reproducibility.

## 2. Materials and Methods

### 2.1. Protein Extraction

Mature maize leaves representing 1.2 g of fresh matter were pooled and ground together in liquid nitrogen using a mortar. To be in standard condition for protein extraction, the resulting powder was aliquoted in 24 tubes, each containing approximately 200 µL of leaf powder. Proteins were extracted using the TCA acetone protocol described in [12]. 

### 2.2. Experimental Design

**Figure 1:**
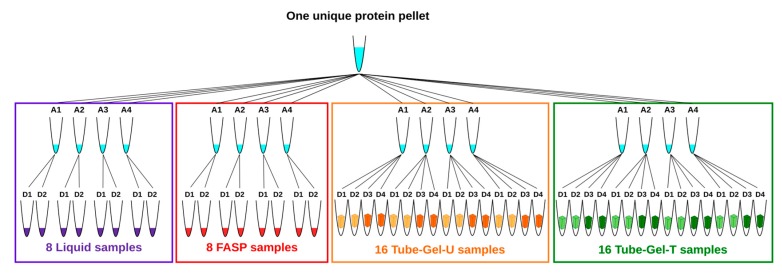
Schema of the experimental design. A single protein pellet was divided into 16 aliquots (A). Proteins were solubilized by using four different methods with four replicates per method. Two independent digestions (D) were performed per protein sample. For the two methods based on tube-gel digestion, two additional digestions were performed on 1 month old gels (dark tube gel symbols).

In order to prevent undesirable variations which could interfere with the sample preparation methods, we used the same starting material for all the methods. For this reason, the 24 protein pellets resulting from extraction were pooled, homogenized, and dried in a speedvac before being aliquoted in 16 tubes that were equally distributed between the methods. Each tube contained between 5.7 mg and 9.4 mg of protein powder. The four tubes attributed to each method represented four independent replicates of solubilization. For the liquid and FASP methods, two independent digestions were subsequently performed for each tube of solubilized proteins. For the two variants of the tube-gel method (tube-gel-U and tube-gel-T), four independent digestions were performed for each tube of solubilized proteins: two of them were performed immediately after gel polymerization, and the other two were performed one month later, in order to evaluate whether in-gel protein conservation affected the MS analysis. At the end, the total number of samples was 48 (Figure 1).

### 2.3. Liquid Digestion Protocol

Proteins were solubilized in 20 µL per mg of extract of a buffer containing 6 M urea, 2 M thiourea, 10 mM dithiothreitol (DTT), 30 mM Tris-HCl pH 8.8, and 0.1% zwitterionic acid labile surfactant (ZALS I, Proteabio, Morgantown, WV, USA) in water. Protein powders were mixed in the buffer using a metal spatula, before vortexing the tubes for 3 min. Samples were centrifuged (14,000 *g*, 25 min, 25 °C) and supernatants were transferred into new tubes. Protein concentrations were estimated using the plusOne 2DQuant Kit (GE Healthcare, Little Chalfont, UK), and adjusted to 1.5 µg·µL^−1^ prior to digestion.

Digestion was performed in 0.2 mL strip tubes from 10 µL of diluted proteins. The tubes were incubated for 30 min at room temperature for protein reduction by the 10 mM DTT present in the solubilization buffer. Then, 2 µL of a solution containing 300 mM of iodoacetamide (IAA) in 50 mM ammonium bicarbonate (BICA) were added, and proteins were alkylated by 1 h incubation at room temperature in the dark. After adding 90 µL of 50 mM of BICA, proteins were digested overnight with 600 ng of trypsin (Promega V5111, Promega, Fitchburg, WI, USA) at 37 °C. Trypsin digestion was stopped by adding 6 µL of 18.6% trifluoroacetic acid (TFA) representing 1% final concentration. Samples were incubated 1 h at room temperature to allow TFA to cleave ZALS I. 

Peptides were desalted using C18 solid phase extraction (SPE) cartridges (strata XL 100 µm ref 8E-S043-TGB, Phenomenex, Torrance, CA, USA) as follows. The samples were first diluted in 2% acetonitrile (ACN), and 0.06% acetic acid in water (washing buffer), up to a final volume of 500 µL. Then, the cartridges were conditioned with 500 µL of ACN and rinsed three times with 500 µL of washing buffer before loading the samples. Peptides were rinsed four times with 500 µL of washing buffer, and eluted two steps by adding 300 µL of 70% ACN and 0.06% acetic acid in water (final pH around 2). Finally, eluted peptides were dried in a speedvac.

### 2.4. FASP Protocol

Proteins were solubilized in 20 µL per mg of extract of a Laemmli-based buffer [13] containing 2% SDS, 60 mM DTT, and 62.5 mM Tris-HCl pH 6.8 in water. After mixing with a metal spatula and short vortexing, samples were heated to 90 °C for 10 min, cooled on ice, and centrifuged (14,000 *g*, 25 min, 25 °C). Supernatants were transferred into new tubes. Protein concentrations were estimated using the plusOne 2DQuant Kit (GE Healthcare), and adjusted to 1.5 µg·µL^−1^ prior to digestion.

Digestion was performed in YM-30 Microcon tubes (Millipore Cat. No. 42410, Millipore, Burlington, MA, USA) on 10 µL of diluted proteins by using the FASP protocol variant 2 [1] with 600 ng of trypsin. Peptide desalting was performed as described above.

### 2.5. Tube Gel with SDS Tris Buffer Protocol

Proteins were solubilized in 20 µL per mg of extract of a buffer containing 3% SDS and 100 mM Tris-HCl pH 8.8 in water. As in Muller et al. [5], the solubilization buffer contained no reducing agent, as it would have negative effects on gel polymerization, and would necessitate an increase in ammonium persulfate (APS) concentration. As a consequence, the rate of peptide oxidation would be increased, which is not desirable. After mixing with a metal spatula and short vortexing, samples were heated to 90 °C for 10 min, cooled on ice, and centrifuged (14,000 *g*, 25 min, 25 °C). Supernatants were transferred into new tubes. Protein concentrations were estimated using the plusOne 2DQuant Kit (GE Healthcare) adjusted to 1.5 µg·µL^−1^ prior to digestion. Gels were prepared in 0.2 mL strip tubes by mixing 10 µL of diluted proteins to 5 µL of a solution of acrylamide/bis-acrylamide (37.5:1) at a concentration of 40%. After short vortexing, 2 µL of freshly prepared APS at 1.75% and 3 µL of TEMED freshly diluted at 10% were added. After short vortexing and spinning in a centrifuge, the tubes were incubated 1 h in the dark for polymerization. Gels were then fixed by two successive baths in 100 µL of 10% acetic acid and 40% ethanol in water, and dried in 96% ethanol for 30 min. 

Proteins were in-gel digested with 600 ng of trypsin, according to the protocol described in [14]. Briefly, gels were rinsed twice with 50 µL of a washing buffer containing 50 mM of BICA and ACN in 1:1 proportion. They were subsequently dried with 50 µL of ACN. Reduction was performed using 50 µL of a solution containing 10 mM of DTT in 50 mM BICA at 56 °C for 45 min. Alkyation was performed using 50 µL of a solution containing 50 mM iodoacetamide for 45 min at room temperature in the dark. Gels were then rinsed and dried as previously mentioned. Gel pieces were rehydrated on ice with 20 µL of trypsin diluted in 50 mM BICA. Digestion was performed overnight at 37 °C. After adding 10 µL of 50 mM BICA, peptides were extracted in two steps with 2 × 50 µL of 0.5% TFA and 50% ACN in water, and transferred into new tubes. Gels were dried with ACN, and the supernatants were transferred to the peptide tubes. Peptides were finally dried in a speedvac.

### 2.6. Tube Gel with Urea SDS BICA Buffer Protocol

Proteins were solubilized at room temperature in 20 µL per mg of extract of a buffer containing 8 M urea, 3% SDS, and 100 mM BICA in water. As previously mentioned, the solubilization buffer for tube gel contained no reducing agent. The rest of the protocol was the same as for tube gel with SDS Tris buffer, except that samples were not heated.

### 2.7. Mass Spectrometry

A total of 48 protein digests (Figure 1) were analyzed on an Eksigent nlc425 nanoHPLC (SCIEX) coupled with a Qexactive+ mass spectrometer (Thermo, Waltham, MA, USA). Peptides were solubilized in 150 µL of a loading buffer containing 2% ACN and 0.1% formic acid (FA) in water. 

For each injection, 4 µL (400 ng) of solubilized peptides were loaded onto a Biosphere C18 pre-column (particle size: 5 μm, pore size: 12 nm, inner/outer diameters: 360/100 μm, length: 20 mm; NanoSeparations, Nieuwkoop, Netherlands) and desalted for 4 min with the loading buffer at 7.5 μL·min^−1^. Peptides were then separated on a Biosphere C18 column (particle size: 3 μm, pore size: 12 nm, inner/outer diameters: 360/75 μm, length: 300 mm; NanoSeparations) using buffer A (0.1% FA in water) and buffer B (0.1% FA in ACN) at 300 nL·min^−1^ as follows: (i) the column was equilibrated during 9 min with 95% of buffer A and 5% of buffer B; (ii) a linear gradient from 95% of buffer A and 5% of buffer B to 65% of buffer A and 35% of buffer B was applied for 75 min; (iii) the column was regenerated with 5% of buffer A and 95% of buffer B for 5 min. Electrospray ionization was performed at 1.8 kV with an uncoated capillary probe (non-coated capillary silica tips, 360/20-10, New Objective Inc., Woburn, MA, USA ). S-lens RF level was set to 50. 

Data were acquired with Xcalibur v4.0 with the following data dependent steps: (1) full MS scan: 75,000 resolution, 350-1400 *m*/*z* mass range, AGC target to 3 × 10^6^, max injection time of 250 ms; (2) MS/MS scan: 17,500 resolution, AGC Target to 1 × 10^5^, max injection time 120 ms, isolation window 1.5 *m*/*z*, normalized collision energy 27. Step 2 was repeated for the eight most intense ions detected in step 1 with the following criteria: minimum threshold 8.3 × 10^3^, precursor charge step of 2 and 3, dynamic exclusion of 50 s, peptide match on and exclusion of isotopes. Raw data were transformed to mzXML format using msconvert (proteowizard 3.0.7069, [15]). 

### 2.8. Protein Identification and Peptide Quantification

Data were searched with X!Tandem (version 2015.04.01.1 [16]) against the maize genome database V5a (https://ftp.maizegdb.org/MaizeGDB/FTP/) and a homemade database containing standard contaminants. Trypsin digestion was set in strict mode with one authorized missed cleavage. Cysteine carbamidomethylation was set as a fixed modification. Methionine oxidation, protein Nter acetylation with or without excision of methionine, Nter glutamine deamidation, Nter carbamidomethyl cysteine deamidation, and Nter glutamic acid dehydration were set as potential modifications. For tube-gel samples, cysteine propionamidation was added as an alternative fixed modification. To allow the identification of additional peptides for proteins identified after this first pass, all samples were submitted to a second pass (refine mode of X!Tandem) in which five missed cleavages were authorized, and tryptophane oxidation, glutamine, and asparagine deamidation were added as potential modifications. For tube-gel samples, lysine and peptide Nter propionamidation were also added as potential modifications during the second pass. Protein inference was performed by using X!TandemPipeline v3.4.3 [17] with the following parameters: peptide E-value less than 0.01, protein E-value less than 10^−5^, one identified peptide by protein. Inference was performed using all samples together. Using a reverse version of the maize protein database as a decoy, the FDR was estimated by X!Tandem to 0.14% and 0.12% for peptide-spectrum match and protein identification, respectively.

Peptide quantification was performed on extracted ion currents (XIC) by using Masschroq 2.2.2 [18] with quantification of 80% of the theoretical natural isotopic profile. Only the most intense isotope was considered for peptide quantification. Identification and quantification data are publicly available at http://moulon.inra.fr/protic/optimized_tube_gel.

### 2.9. Data Analysis

To quantify proteins, we used two complementary approaches. We first used XIC to quantify proteins based on peptide intensities. Only protein-specific peptides present in at least 85% of the injections and showing a significant correlation (r > 0.5) with the other peptides of the same protein were kept for further data analysis. Normalization was performed, taking into account peptide retention time as described in [19]. Only proteins quantified with at least two peptides were considered. As a result of this selection, 899 proteins were quantified based on XIC. Protein relative abundances were computed as the sum of the normalized values of the selected peptides. One-way analyses of variance were performed on log_10_-transformed abundance values. The resulting *p*-values were adjusted for multiple comparisons [20]. Proteins with adjusted *p*-value < 0.01 were considered as showing significant abundance variations. 

To analyze qualitative variations, we also used spectral counts, removing the proteins that produced less than three spectra in any sample. We indeed considered that these proteins were of too low abundance to be reliably quantified. This lead us to reduce the initial dataset containing 2966 identified proteins to 1358 proteins. To identify proteins showing significant spectral counts variations, we performed a general linear model, and we used the same criteria of significance as for the XIC-based approach. The Self Organizing Tree Algorithm (SOTA [21]) was used to cluster proteins according to their abundance or spectral count profile. All data analysis was performed by using the R package (v3.3.3) [22].

### 2.10. Transmembrane Regions Prediction

Transmembrane regions were predicted by using the Phobius [23] and TMHMM2.0 [24] algorithms. A protein was considered as membrane associated when domains with at least 10 amino acids were found by both algorithms.

## 3. Results

In the perspective of standardizing the use the tube-gel method for large-scale experiments, we developed a miniaturized version with two variants based on different buffers. To evaluate the quality of the resulting data, we compared the performances of these two variants to those of two other commonly used sample preparation methods, liquid and FASP, using the sample protein pellet and the same amounts of proteins and trypsin. Solubilization and digestion were performed independently for each method as described below:

1. Tube-gel-U method: protein solubilization in a buffer containing mainly urea and SDS, protein trapping by acrylamide polymerization in tubes, gel rinsing, in-gel trypsinolysis, and recovery of the peptide mix by rinsing with ACN/water;
2. Tube-gel-T method: protein solubilization in a Tris-SDS buffer, heating at 90 °C for 10 min, protein trapping by acrylamide polymerization in tubes, gel rinsing, in-gel trypsinolysis, and recovery of the peptide mix by rinsing gels with ACN/water;
3. Liquid method: protein solubilization in a buffer containing mainly urea, thiourea and ZALS I detergent, dilution with BICA, trypsinolysis, and cleaning of the peptide mix by SPE;
4. FASP method: protein solubilization in a Laemmli buffer, concentration of proteins on a filtration device, replacement of the SDS buffer with a urea buffer, dilution with BICA, trypsinolysis, recovery of the peptide mix under the filter, and cleaning by SPE.

To test, more thoroughly, the robustness of the tube-gel method, we also analyzed the stability of in-gel protein storage over time. To do so, trypsinolysis was performed either on freshly prepared gels (TG-Unew and TG-Tnew) or on one month-old gels that were stored at 4 °C (TG-Uold and TG-Told).

For each method, four independent solubilizations and two to four independent digestions per solubilization were performed (Figure 1), so that the total number of injected samples was 48. For two of them the number of assigned spectra was approximately half that of the other samples. A third one was identified as an outlier on the representation of the principal component analysis (PCA) based on estimated protein abundances (Figure S1). Because these three samples corresponded to three different methods, we assumed that they reflected technical issues, rather than differences between methods. For this reason, we decided to discard them from the final analysis.

### 3.1. Tube Gel Miniaturization

To make sample preparation more convenient for large-scale experiments, we first developed a miniaturized version of the tube-gel method. To this end, we adapted the method to 0.2 mL strip tubes. This allowed to digest volumes of 20 µL without any manipulation of the gel itself: no gel cutting into smaller pieces, and no manual operation to unstick the gels from their support were required. In addition, all operations were performed in the same tube, using a dispenser to pour gel solutions, APS and TEMED in the tubes, and a multichannel pipette to recover peptides after digestion. 

In terms of costs, the tube-gel method was the least expensive: 137 € for a 96-well plate, versus 729 € and 711 € for the FASP and liquid methods, respectively. For the tube-gel method, the dispenser was the most expensive consumable, while for the FASP and liquid methods, the most expensive consumables were ZALS I and filters, respectively.

In terms of time, preparing 48 samples (without taking into account the overnight digestion) took around 13 h for the tube-gel method, and 15 h for the FASP and liquid methods. The time difference in favor of the tube-gel method is due to a shorter drying time (4 h vs. 10 h). The volume of buffer to eliminate was indeed much higher for the FASP and liquid methods than for the tube-gel method (600 µL vs. 150 µL, respectively). However, this time gain was partially offset by the preparation of the gels, which took 4 h.

In terms of flexibility, the tube-gel method allowed the sample preparation to be split into a higher number of steps than the FASP and liquid methods. For example, we could interrupt the manipulations after the gel preparation or just before the digestion, which was impossible with the FASP method, and not recommended with the liquid method. In addition, the FASP and liquid method both required a 2 h-peptide desalting step, that required the permanent presence of the manipulator at the bench. In this regard, the tube-gel method thus proved to be simpler to implement than the FASP and the liquid methods.

### 3.2. Protein Extraction

**Figure 2:**
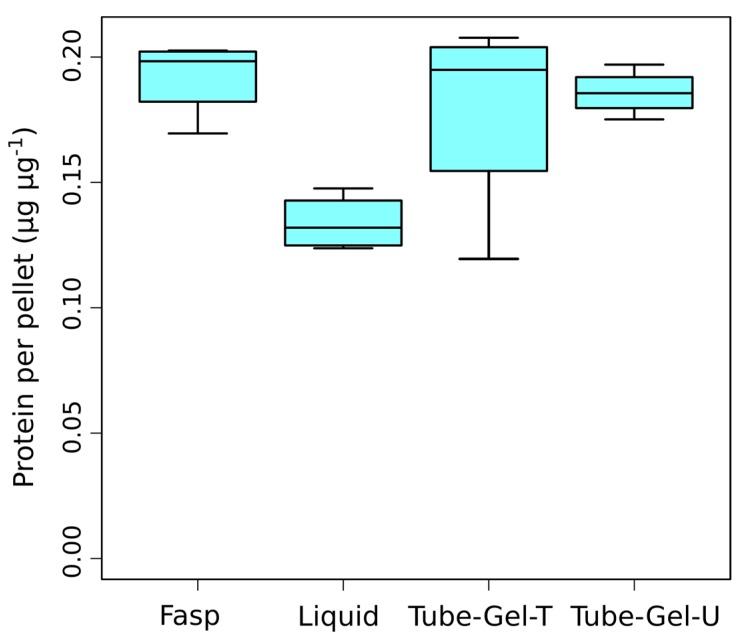
Protein yields obtained with the four tested methods of sample preparation.

We first examined to which extent the different solubilization buffers used in the four sample preparation methods affected the protein extraction yield. Based on the protein dosage performed after solubilization, we observed that the three SDS-based methods (FASP, tube-gel-U and tube-gel-T) had similar protein yields, with means between 0.18 µg and 0.19 µg of protein per µg of pellet (Figure 2). This result indicates that heating the solubilization solution in the tube-gel-T method did not provide any advantage compared to the tube-gel-U method, in which solubilization was performed at room temperature. The liquid method had a significantly lower protein yield than the other methods (0.13 µg·µg^−1^, *p*-value = 0.014, Figure 2), confirming that SDS is beneficial to protein extraction.

### 3.3. Peptide Identification

**Figure 3:**
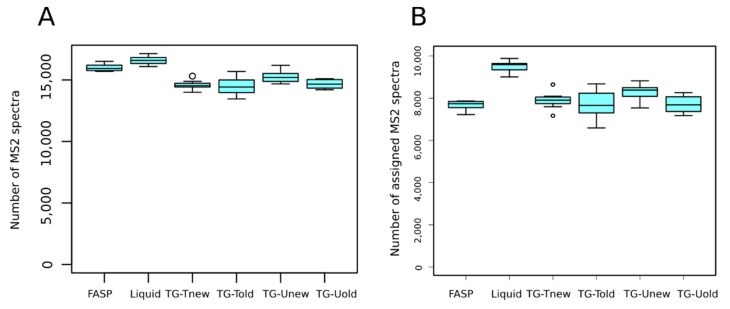
Numbers of MS^2^ spectra (**A**) and of assigned spectra (**B**) obtained for FASP, liquid digestion and the variants of the tube-gel methods.

On average, 15,223 MS^2^ spectra per sample were generated. We detected a significant effect of the sample preparation method on the number of MS^2^ spectra per sample (*p*-value < 0.001). Indeed, the liquid and FASP methods produced significantly more MS^2^ spectra than the tube-gel methods (Figure 3A). Whatever the method, almost all MS^2^ spectra were selected by X!Tandem for database search (98.5 to 99.2%, not shown). This indicates that the sample preparation method did not affect the quality of spectra.

On average, 8109 (53.3%) MS^2^ spectra were assigned to peptides. We detected a significant effect of the method on the number of assigned MS^2^ spectra (*p*-value < 0.001), due to a higher value for the liquid method than for the others (9494 vs. 7710 to 8289, respectively; Figure 3B). We also detected that the liquid method identified significantly more unique peptides than the other methods (on average 7553 vs. 5996 to 6463, respectively).

The percentage of miscleaved peptides ranked from 12% with FASP to 17% with the liquid method. It varied between 13% and 15% with the tube-gel methods.

### 3.4. Protein Qualitative Variations

**Figure 4:**
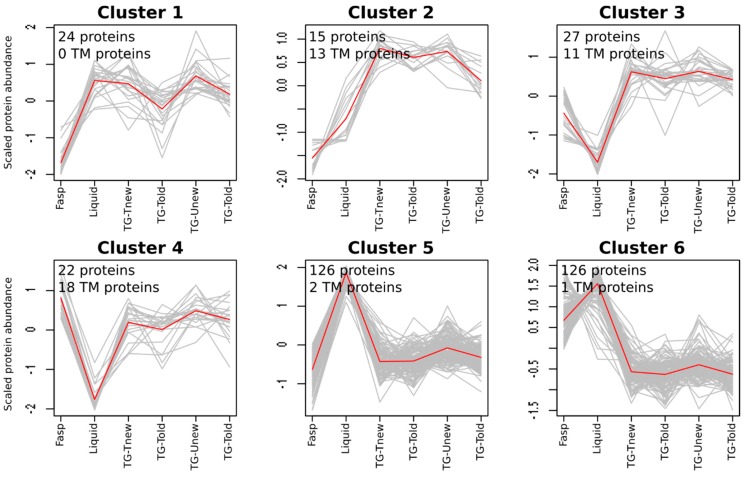
Protein clustering according to spectral counts. Data were scaled: 0 corresponds to the general mean of each protein in all samples.

To determine whether the methods extracted specific sets of proteins, we analyzed the protein qualitative variations. To do so, we analyzed the spectral counting data using a generalized linear model to identify proteins showing significant spectral count variations in response to the method. The TG-Uold, TG-Unew, TG-Told, and TG-Tnew were considered as different methods. For 340 proteins out of 1358, the methods significantly affected the number of spectra (adjusted *p*-value < 0.05). These proteins were grouped in six clusters by a SOTA analysis (Figure 4, Table S1).

Clusters 5 and 6 (126 proteins each) were the two main clusters. They contained proteins for which the liquid method provided the highest numbers of spectra, which is consistent with the higher number of identified peptides observed with this method. By contrast, clusters 3 and 4 (27 and 22 proteins, respectively) gathered proteins for which the liquid method provided the lowest numbers of spectra. Clusters 1 and 2 (24 and 15 proteins, respectively) gathered proteins for which the tube-gel method provided more spectra than the FASP method, the liquid method being similar to the tube-gel method (cluster 1), or intermediary between the FASP and tube-gel methods (cluster 2). No cluster clearly separated the different variants of the tube-gel method, indicating that neither the solubilization buffer nor the gel aging affected the composition in proteins.

Since SDS is known to promote the extraction of membrane proteins, we studied the ability of the different sample preparation methods to extract transmembrane proteins (TPs). TPs were defined here as proteins containing a predicted transmembrane domain (see Materials and Methods). In a first approach, we counted the number of TPs identified in at least one sample for each method. The FASP method identified the highest number of TPs (236) and the liquid method the lowest (192), with the tube-gel method being intermediate (208 to 217). The sets of identified TPs were different from one method to another, as the total number of TPs in the dataset was 301. Most of the TPs were minor proteins in terms of abundance: in the complete dataset, the median number of spectra for TPs was 4.2, while it was almost twice (7.8) for the other proteins. For this reason, only 99 TPs were retained for the spectral count analysis. For most of them (60), the sample preparation methods had no significant effect on the number of spectra. This is probably because the statistical power was too low to detect significant variations for proteins quantified with few spectra. The remaining 39 TPs were mostly classified in clusters 2, 3, and 4 (Figure 4), where they represented the majority of the proteins. Two of these clusters (3 and 4) gathered proteins with a lower number of spectra in the liquid method than in the other methods. This is consistent with the fact that the liquid method is the only one where no SDS was used during protein solubilization. The other clusters gathered a low number of TPs (0 to 2).

### 3.5. Protein Quantification

**Figure 5:**
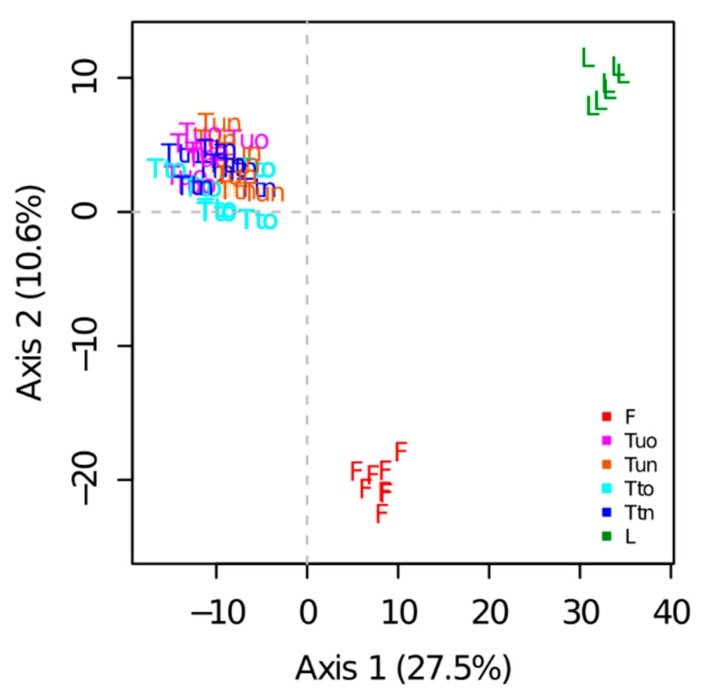
Principal component analysis performed on XIC data. F: Fasp; L: Liquid; Tto: tube-gel-Told; Ttn: tube-gel-Tnew; Tuo: tube-gel-Uold; Tun: tube-gel-Unew.

A total of 899 proteins were reproducibly quantified based on XICs. PCA performed on the protein abundances estimated from peptide intensities showed that samples were well grouped according to the sample preparation methods (Figure 5). The first principal component (PC1, 27.5% of the variability) separated tube-gel from liquid samples. The second principal component (PC2, 10.6% of the variability) separated FASP samples from the other samples. Samples obtained from the variants of the tube-gel method could not be distinguished on the first plan of the PCA. Nonetheless, they were slightly separated on PCs 3, 4, and 5 (respectively 3.3%, 2.9%, and 2.7% of the variation, not shown).

A one-way analysis of variance indicated a highly significant effect of the sample preparation method on the abundances of 571 proteins. The cluster analysis showed that the liquid method differed from the others (Figure S2): in clusters 2, 5, and 6 (cluster 2 being the largest cluster with 208 proteins), the protein abundances obtained with the liquid method were either the highest (clusters 5 and 6) or the lowest (cluster 2). No cluster highlighted a difference between the variants of the tube-gel method.

Regarding TPs, 87 were reproducibly quantified based on XICs, and 70 of them showed significant abundance variations depending on the sample preparation method. Most of them (83%) were classified in cluster 2, i.e., were less abundant in the liquid samples. Only 24 of the 70 proteins showing a significant abundance variation according to XIC analyses also showed significant variations in their number of spectra, which shows the complementarity between the two quantification approaches: spectral counting allowed the detection of quantitative changes that affected peptide reproducibility, while XIC-based quantification allowed the detection of abundance changes of proteins that were reproducibly detected. 

Another important criterion to discriminate between methods is the technical reproducibility. To evaluate this parameter, the mean coefficients of variation (CVs, standard deviation/mean) were computed for each sample preparation method. The CVs were similar for all the methods: 21.7 and 20.9 for FASP and liquid methods, respectively; between 20.6 and 22.5 for the tube-gel methods. These results indicate that the methods exhibited equivalent technical reproducibility.

### 3.6. Post-Translational Modifications

**Figure 6:**
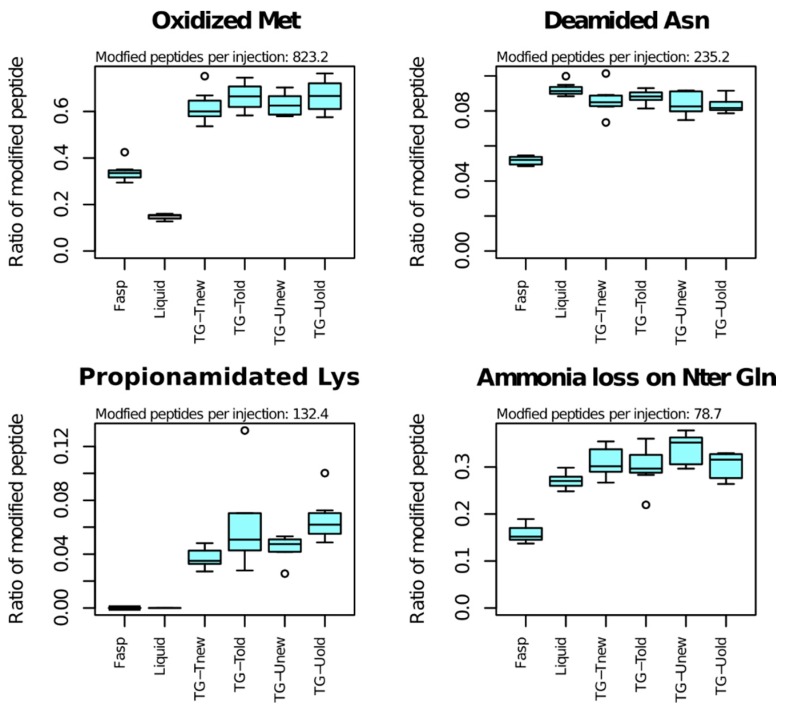
Effect of the methods on PTM frequencies. Only major effects are shown. All graphs are available in Figure S3. Ratio: modified peptides/(modified + unmodified peptides). Modified peptides per injection: mean number of modified peptides per injection.

As the sample preparation methods can differ in a way that can influence peptide PTMs, we examined to which extent the proportions of modified peptides varied across methods (Figure 6 and Table 1).

The most frequent PTM was methionine oxidation, that affected 823 peptides per sample on average (Table 1). Among Met-containing peptides, the proportion of those bearing a Met-oxidation was highly variable across methods: 15% for the liquid method, 34% for the FAST method and 65% for tube-gel methods on average. Oxidation also occurred for tryptophane, but it affected a small number of peptides (33 on average).

The second most frequent PTM was deamidation of asparagine (235 peptides per sample on average). The proportion of Asn-containing peptides bearing Asn-deamidation varied significantly across methods (*p*-value < 10^−10^). The FASP method exhibited the lowest percentage of Asn-deamidation (5%), while the percentage was between 8% and 9% for the liquid and tube-gel methods. Deamidation was also detected for glutamine on a small number of peptides (27 per sample on average), and its proportion did not vary across methods.

Lysine propionamidation was the third most abundant PTM (132 peptides in average). Propionamidation is an oxidation that can occur in presence of acrylamide monomers. This PTM was also detected at low frequency for cysteine and at peptide Nter end in the samples of the four variants of the tube-gel method.

Ammonia loss of N-terminal glutamine affected 79 peptides per sample on average, and the proportion of modified peptides varied highly significantly across methods. The proportion of modified peptides was 16% for the FASP method. It was 27% for the liquid method and 30 to 34% for the tube-gel methods. All the other analyzed PTMs affected less than 50 peptides in average, and showed no significant variation.

## 4. Discussion

In this paper, we aimed at developing a miniaturized version of the tube-gel method convenient for large-scale experiments. Compared to the FASP and liquid methods, our version of the tube-gel method is much less expensive, because only low-cost consumables are specifically needed (acrylamide, strip tubes), while SPE resins and specialized membranes are necessary to implement the liquid and FASP methods, respectively. It is simpler to implement because of the absence of peptide desalting. It is also more flexible because of the possibility to split the sample preparation into several steps and to store proteins in-gel. We indeed showed that the duration between gel preparation and protein digestion had no notable effect on protein identification or quantification. The gain in time to handle 48 samples was quite moderate. However, as we performed tube-gel in 0.2 mL strip tubes, the method can be easily scaled to 96 samples, or to several 96-well plaques, with minimum additional time. This is also the case of the liquid method, but not of FASP, which was limited by the capacity of the centrifuge used at several steps of sample preparation. Therefore, FASP cannot be used to handle more than 48 samples at a time, unless using several centrifuges in parallel. 

Proteins were extracted by using the TCA/acetone method, in which proteins are precipitated with all cellular debris without any prior solubilization. This extraction method is widely used for plant protein extraction, as it allows immediate inhibition of protease activity and solubilization of chlorophyll. In addition, it is well suited for large-scale experiments. The tube-gel, FASP and liquid methods were all tested with their own appropriate solubilization buffer on the same TCA/acetone pellet. In the case of the tube-gel method, we compared two SDS-containing buffers: one was a Tris buffer, that was heated to facilitate protein solubilization; the other was a urea buffer, that could not be heated because of urea degradation. We showed no difference between these two variants of the tube-gel method in terms of protein identification or quantification. However, the tube-gel-U method can be preferred, because of the absence of sample heating, and because polymerization seemed to perform better (less unpolymerized supernatant). FASP was also based on a SDS-containing buffer, while the liquid method was based on a urea buffer. As expected, because SDS allows a better solubilization, a better protein yield was obtained with the three SDS-based methods (tube-gel-U, tube-gel-T and FASP) than with the liquid method. The yield difference was relatively small, and did not justify, per se, the selection of a particular buffer. In addition, we observed that the different methods preferentially extracted or digested different sets of proteins, in particular, regarding membrane proteins which were better represented in the SDS-based methods. This trend was clearly observed in spectral counts, as well as in MS^1^ XICs. Thus, even the transmembrane domain-containing proteins that were reproducibly solubilized by urea-ZALS I were, in general, present in lower abundance with the liquid method than with the tube-gel and FASP methods. In this respect, the liquid method was the most distinct from the others.

Although an equal amount of peptide mix was used for all injections, the liquid method identified more unique peptides than the FASP and tube-gel method. This is in contradiction with the results of Muller et al. [5], probably because of the addition of a detergent (ZALS I) to the solubilization buffer in the present study. Smaller differences were also observed between the FASP and the tube-gel method. The absence of reducing agent in the solubilization buffer used in the tube-gel method could be partially responsible for this difference. However, our experimental design did not allow us to discriminate the effects of the solubilization buffer from those of the digestion method.

Methionine oxidation was the most frequently observed PTM. We showed a significant effect of the sample preparation method on its frequency. The liquid method indeed produced 2 and 4.5 times less oxidized forms than the FASP and tube-gel methods, respectively. This can probably be explained by a longer exposure to air with the FASP and tube-gel methods, and by the presence of APS in tube gels. The variation of tryptophane oxidation, that involved 33 peptides in average, can be explained by the same factors. As expected from the presence of unpolymerized acrylamide in contact with proteins, a relatively large number of peptides (238 in average) were propionamidated on cysteine, lysine, or on their Nter in tube-gel samples. Regarding the other PTMs, the results obtained with the tube-gel methods were similar to those obtained with the liquid method or with both the liquid and FAST methods.

## 5. Conclusions

The sample preparation methods tested in this study have different pros and cons regarding data quality. On one hand, according to the number of identified proteins and to the level of methionine oxidation, the liquid method was superior to all the other methods. On the other hand, the FASP and tube-gel methods better solubilized membrane proteins, due to the presence of SDS. The FASP method showed no particular advantage compared to the tube-gel method, except for the frequency of few PTMs. However, although both of them are based on the solubilization of proteins by SDS, they are not interchangeable, because there are differences in the sets of proteins preferentially extracted or digested.

From an operational standpoint, the tube-gel method as optimized here is certainly the most adaptable to the preparation of large series of samples. It is indeed less expensive, simpler, and more flexible than the FASP and liquid methods. It is also scalable, and the absence of a gel aging effect allows the planning of large-scale experiments by preparing tube gels in advance. One person could then handle the digestion of 400 samples without automation by using four 96-well plates. A small time shift between their processing for reduction, alkylation, and trypsinolysis would be necessary to ensure equal incubation time for all samples, while drying could be performed simultaneously in a speedvac accepting four plates. Moreover, data quality was acceptable compared to the FASP and liquid methods. The fact that less proteins were detected with the tube-gel method than with the liquid method can easily be compensated by the injection of a higher amount of the peptide mix. The high levels of methionine oxidation cannot be compensated, but Met-containing peptides are already known to be unstable, and not recommended for protein quantification. In the end, we consider that the differences observed in the MS results are offset by the advantages in terms of time, simplicity and consumable costs. For this reason, we recommend the use of the tube-gel method for large-scale experiments.

## Figures and Tables

**Table 1:** Post-translational modification (PTM) frequencies.

	FASP	Liquid	TG-Tnew	TG-Told	TG-Unew	TG-Uold	Mean Number of Modified Peptides	*p*-Value (Chi-Square)
Met oxidation	33.97%	14.71%	61.75%	66.38%	62.98%	66.7%	823	0
Asn deamidation	5.17%	9.24%	8.6%	8.81%	8.42%	8.31%	235	1.54 × 10^−7^
Lys propionamidation	0.00%	0.00%	3.7%	6.24%	4.49%	6.55%	132	1.73 × 10^−106^
Nter Gln ammonia loss	15.77%	27.1%	31.01%	30.07%	33.96%	30.42%	79	4.27 × 10^−5^
Nter acetylation	3.37%	3.08%	2.56%	2.47%	2.54%	2.38%	66	0.203
Nter propionamidation	0.00%	0.00%	1.16%	1.26%	1.65%	1.46%	59	5.51 × 10^−42^
Cys propionamidation	0.00%	0.00%	6.26%	7.46%	6.35%	7.18%	46	5.68 × 10^−27^
Trp oxidation	4.4%	2.51%	4.31%	4.58%	4.17%	5.55%	33	0.042
Gln deamidation	0.75%	0.87%	1.03%	1.1%	1.34%	1.14%	27	0.337
Nter carbamidomethylated Cys ammonia loss	13.31%	36.00%	29.58%	30.75%	32.72%	30.82%	22	0.051
Nter Glu water loss	2.33%	3.49%	2.85%	2.42%	2.83%	2.72%	12	0.919

## Supplementary Materials

The following are available online at http://www.mdpi.com/2227-7382/6/1/6/s1, Figure S1: Quality control PCA on XIC data, Figure S2: Clustering according to XIC data; Figure S3: PTM frequencies; Table S1: proteins belonging to the clusters shown in Figure 4.
