# Supplementary material for: Evaluation of Optimized Tube-Gel Methods of Sample Preparation for Large-Scale Plant Proteomics
Source: Proteomes. 2018 Jan 30;6(1):6. doi: 10.3390/proteomes6010006 (PMC5874765; doi:10.3390/proteomes6010006)
Supplement: Supplementary file 1 [file proteomes-06-00006-s001.zip › Figure_S3.pdf]

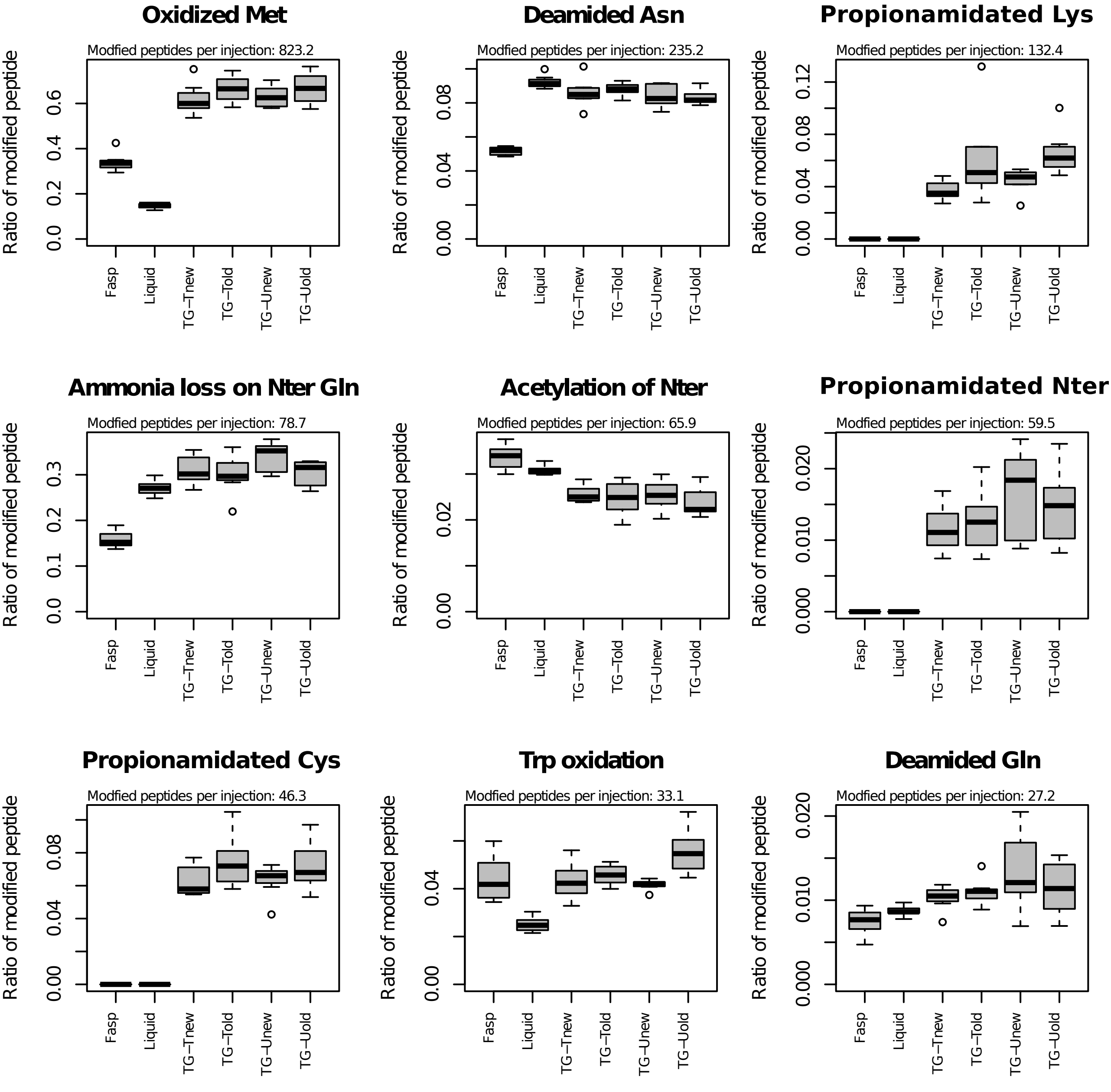
Oxidized Met
Modfied peptides per injection: 823.2
Deamided Asn
Modfied peptides per injection: 235.2
Propionamidated Lys
Modfied peptides per injection: 132.4
Ammonia loss on Nter Gln
Modfied peptides per injection: 78.7
Acetylation of Nter
Modfied peptides per injection: 65.9
Propionamidated Nter
Modfied peptides per injection: 59.5
Propionamidated Cys
Modfied peptides per injection: 46.3
Trp oxidation
Modfied peptides per injection: 33.1
Deamided Gln
Modfied peptides per injection: 27.2
Ratio of modified peptide
Fasp
Liquid
TG−Tnew
TG−Told
TG−Unew
TG−Uold

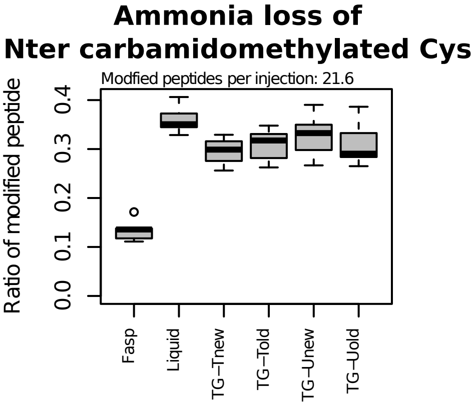
Ammonia loss of
Nter carbamidomethylated Cys
Modfied peptides per injection: 21.6
Ratio of modified peptide
0.0
0.1
0.2
0.3
0.4
Fasp
Liquid
TG−Tnew
TG−Told
TG−Unew
TG−Uold

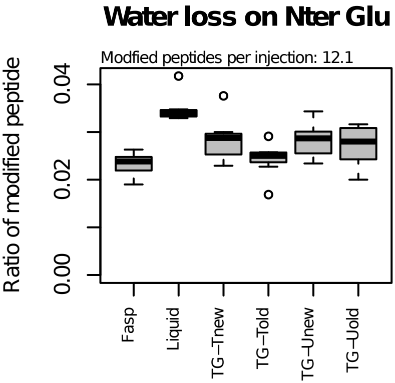
Water loss on Nter Glu
Modfied peptides per injection: 12.1
Ratio of modified peptide
0.00
0.02
0.04
Fasp
Liquid
TG−Tnew
TG−Told
TG−Unew
TG−Uold
