# Supplementary figures and images for: Evaluation of Optimized Tube-Gel Methods of Sample Preparation for Large-Scale Plant Proteomics
Source: Proteomes. 2018 Jan 30;6(1):6. doi: 10.3390/proteomes6010006 (PMC5874765; doi:10.3390/proteomes6010006)

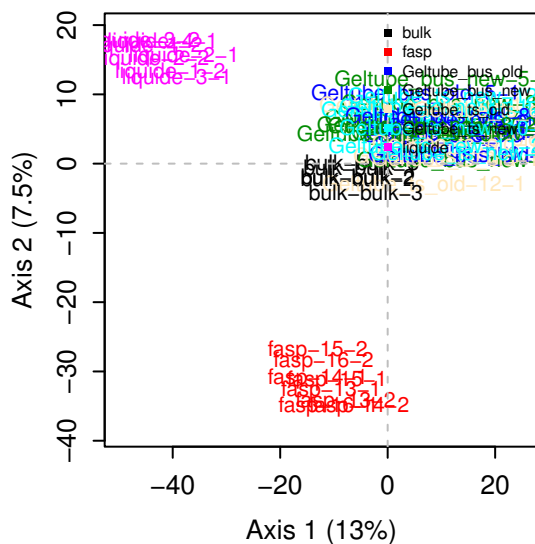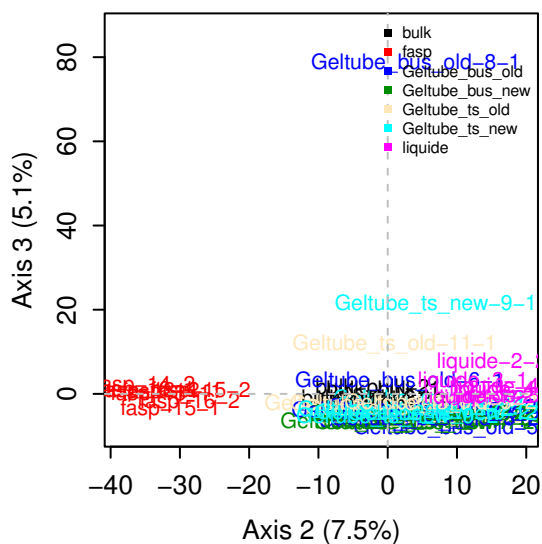

**Figure S1**  
Quality control PCA on XIC data. Outlier detected on Axis 3

Supplement: Supplementary file 1 [file proteomes-06-00006-s001.zip › Figure_S1.pdf]

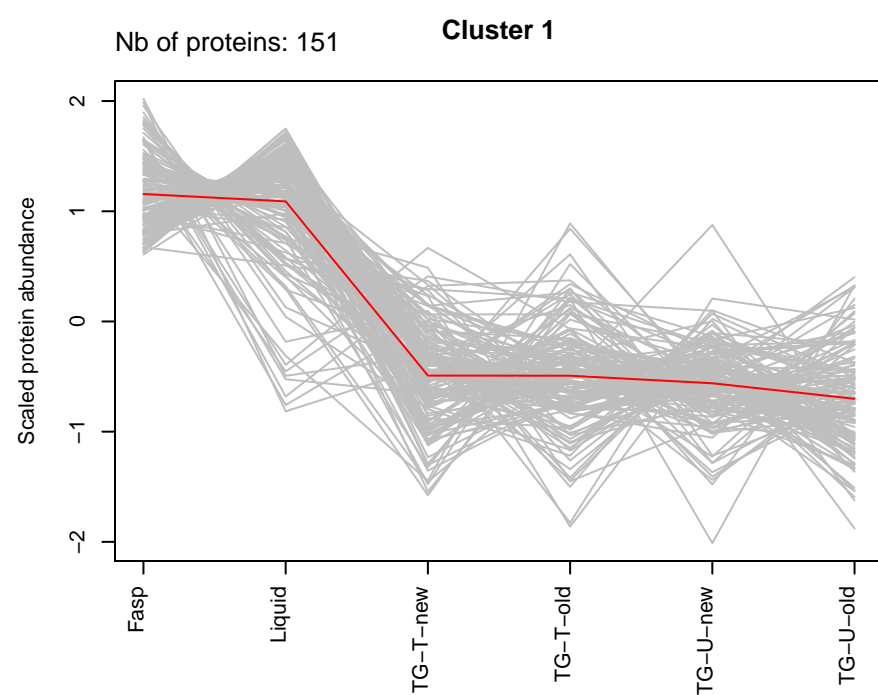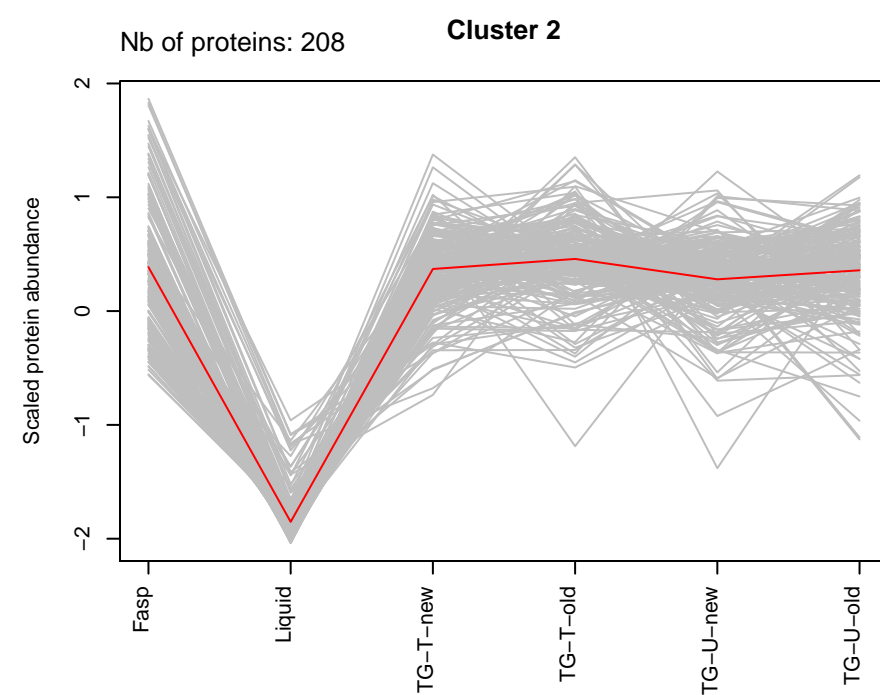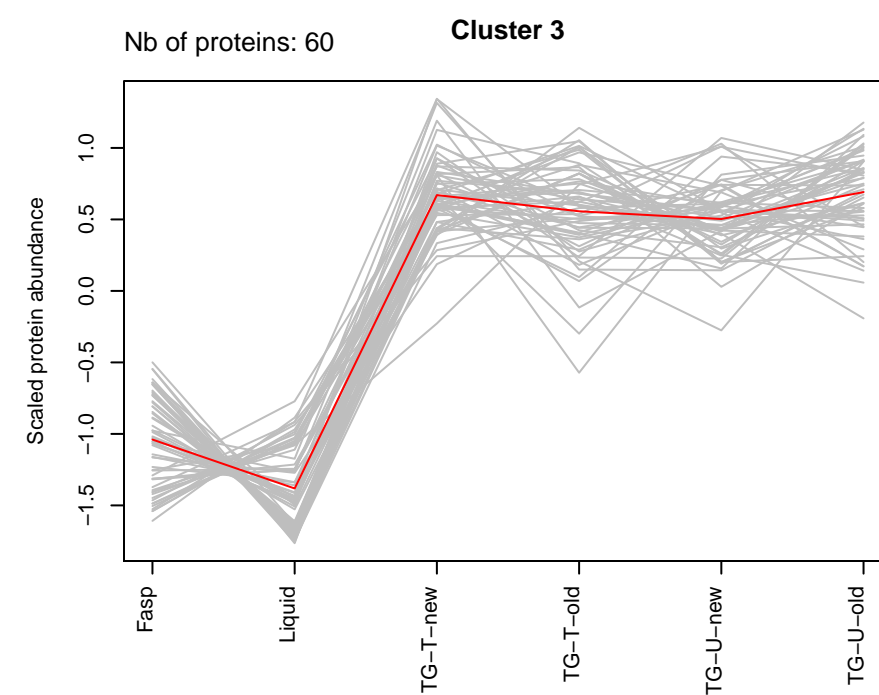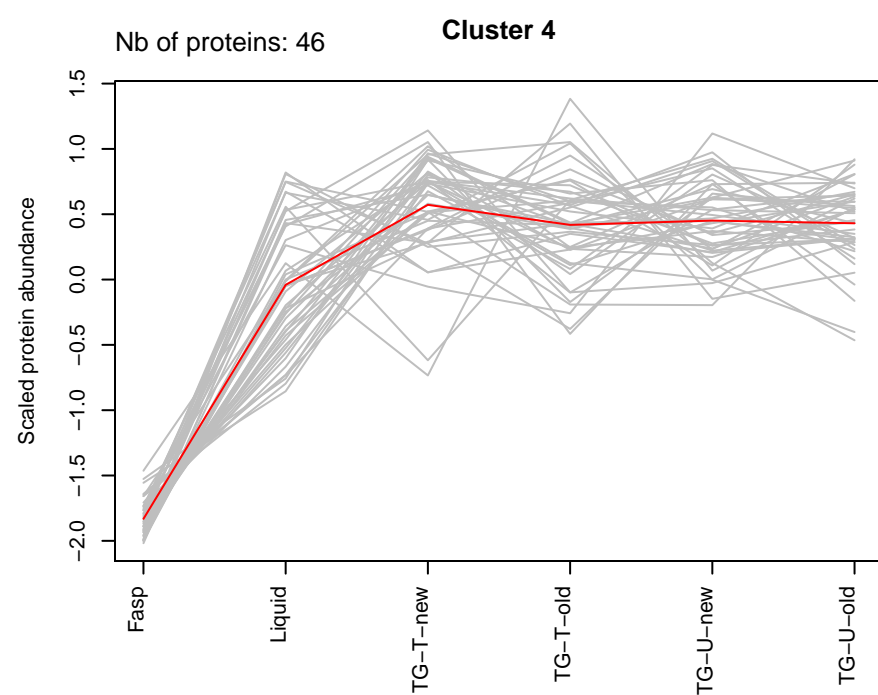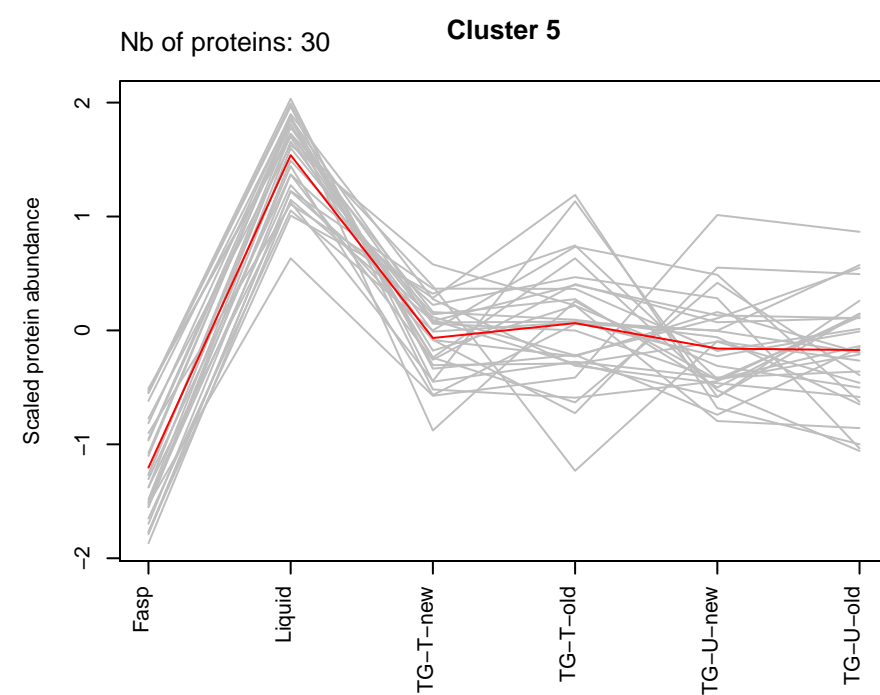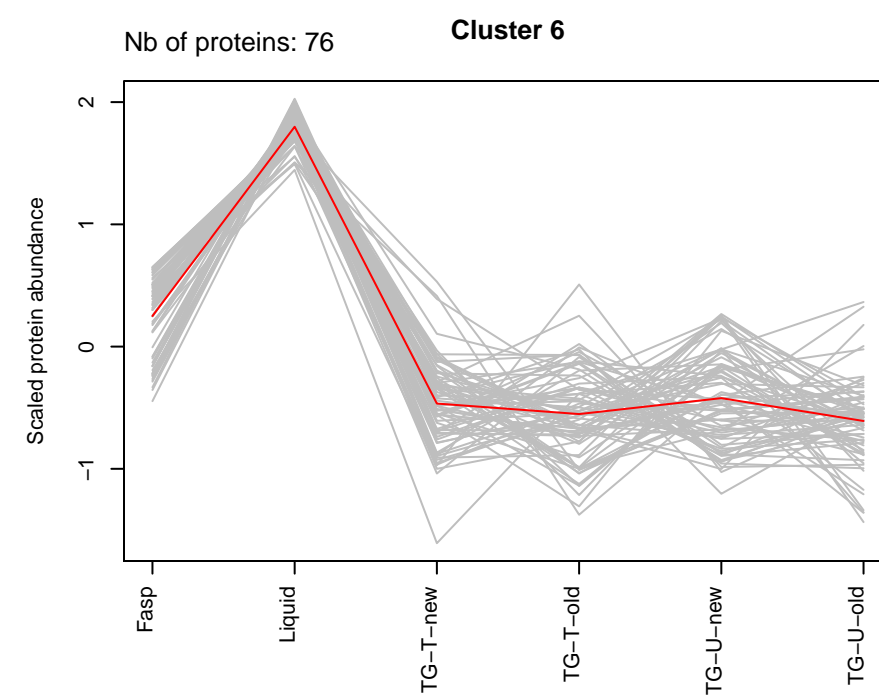

Supplement: Supplementary file 1 [file proteomes-06-00006-s001.zip › Figure_S2.pdf]
